# Keratin 5 marks cancer-propagating cells sustained by an osteopontin-producing niche in high-grade serous ovarian carcinoma

**DOI:** 10.47248/chp2603020005

**Published:** 2026-04-25

**Authors:** Mallikarjun Bidarimath, Coulter Q. Ralston, Nandini Bidarimath, Ian M. Rose, Darianna Colina, Elisa Schmoeckel, Andrew K. Godwin, Doris Mayr, Lora H. Ellenson, Andrea Flesken-Nikitin, Alexander Yu. Nikitin

**Affiliations:** 1. Department of Biomedical and Translational Sciences, Cornell University, Ithaca, NY 14853, USA; 2. Meinig School of Biomedical Engineering, Cornell University, Ithaca, NY 14853, USA; 3. Institute of Pathology, Ludwig Maximilians University, Munich 80337, Germany; 4. Pathology & Laboratory Medicine, University of Kansas Medical Center, Kansas City, KS 66160, USA; 5. Memorial Sloan Kettering Cancer Center, NY 10065, USA

**Keywords:** Cancer cell niche, cancer heterogeneity, ovarian carcinoma, chemoresistance

## Abstract

High-grade serous carcinoma (HGSC) is the most common and aggressive form of ovarian cancer. Advanced HGSCs exhibit pronounced cellular heterogeneity, including a subset of cancer-propagating cells (CPCs, also known as cancer stem cells) that are highly tumorigenic and display stem cell-associated properties such as self-renewal and chemoresistance. In contrast, a substantial fraction of HGSC cells is non-tumorigenic. The role of these non-cancer-propagating cells (non-CPCs) and their relationship to CPCs remain poorly understood. Here, we demonstrate that neoplastic cells expressing the intermediate filament protein keratin 5 (KRT5) represent bona fide CPCs. KRT5+ cells form cancer organoids over successive passages, are tumorigenic in serial dilution xenograft assays, and are resistant to the antineoplastic agents, doxorubicin and cisplatin. Single-cell lineage-tracing experiments show that KRT5+ CPCs give rise to KRT5− cells. KRT5+ and KRT5− populations exhibit distinct gene expression profiles, with KRT5− cells characterized by expression of SPP1, which encodes the secreted factor osteopontin (OPN). Treatment with OPN enhances HGSC organoid growth and chemoresistance, whereas SPP1 knockdown reverses these effects. Together, these findings support a model in which HGSC contains two hierarchically related cell populations: KRT5+, OPN-responsive CPCs and KRT5−, non-tumorigenic cells that form a niche producing OPN. Inhibiting pathways that sustain this niche may enable reduced dosing of highly toxic chemotherapeutic agents while enhancing therapeutic efficacy in HGSC.

## 1. Introduction

Ovarian/extra-uterine high-grade serous carcinoma (HGSC) is the most common and aggressive type of ovarian cancer [1,2]. It often has no symptoms at early stages and over 80% of patients are diagnosed at advanced, usually incurable, cancer stages, when the tumors have already metastasized [3,4]. The overall 5-year survival rate of patients with advanced epithelial ovarian cancer is 32% [5]. Standard chemotherapy can substantially reduce the size of the tumor and transiently improve patients’ health status. However, due to extensive cellular heterogeneity, a fraction of cancer cells evades chemotherapy and advances the disease progression after a brief period of dormancy [6–8].

The evolution of cancer cells and the mechanisms responsible for the development of chemoresistance remain insufficiently elucidated. Some cancer cells are highly tumorigenic and are called cancer-propagating cells (CPCs), cancer stem cells or cancer-initiating cells [2,9–12]. These cells feature stem cell-associated properties, such as self-renewal and chemoresistance. Several markers of CPCs, such as CD44, CD24 and CD133, have been reported [13–15]. However, their prognostic value and the stability of the associated phenotypes remain debatable [11,16–18].

At the same time, a significant fraction of cancer cells, in many cases the majority, has low, if any, tumorigenicity. The role of such non-cancer-propagating cells (non-CPCs) in HGSC progression and chemoresistance, and cellular dynamics between CPCs and non-CPCs remain largely unknown. Non-CPCs can be considered a byproduct of stem cell-like ability of CPCs to differentiate. However, non-CPCs may also have niche-like properties typical of normal tissue homeostasis [9,19–21].

Previously it has been reported that epithelial ovarian cancer cell lines expressing the intermediate filament protein keratin 5 (KRT5) are more resistant to cisplatin-induced apoptosis [22]. Furthermore, it has been suggested that KRT5 overexpression is associated with serous ovarian cancer recurrence and chemotherapy resistance [23]. However, it remains unknown whether KRT5+ cells represent CPCs.

In this study, we found that KRT5+ cells show features typical for CPCs, such as high organoid forming capacity in consecutive passages, tumorigenicity in serial dilutions, the ability to generate KRT5− progeny, and chemoresistance. Notably, growth and chemoresistance of KRT5+ CPCs is sustained by osteopontin (OPN) produced by non-CPCs, a secreted, sialic acid-rich, glycosylated phosphoprotein encoded by the *SPP1* gene [24].

## 2. Results

### 2.1. Clinical relevance of KRT5+ cells in HGSC

Previous studies reported presence of KRT5+ cells in OV432, SKOV3, OVCAR3, PEO1, and PEO4 cell lines at frequencies ranging from 2.4% to 52.7% [22]. Consistent with this observation, we observed a subpopulation of KRT5+ cells in cell lines CAOV-3 (1.82%), CAOV-4 (4.99%) and SKOV3 (7.91%) (Table S1).

**Figure 1.**
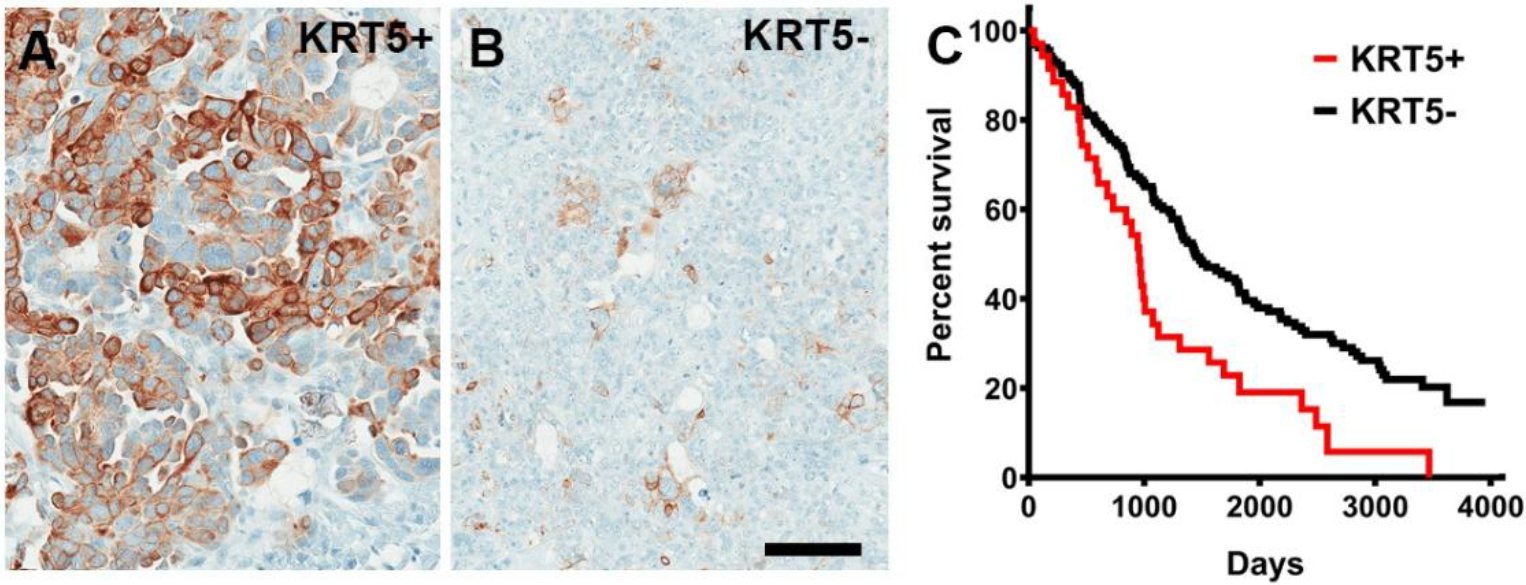
KRT5+ cells in HGSC. **(A, B)** High (A, KRT5+, ≥40% stained neoplastic cells, n = 37) and low (B, KRT5−, n = 160) frequency of KRT5 expressing cancer cells. **(C)** Kaplan-Meier survival analysis of HGSC patients stratified according to frequency of KRT5 expressing cells (>40% of labeled cells, KRT5+ *vs*. KRT5−, 955 days *vs*. 1452 days, Log-rank *P* = 0.0015). A and B, Elite ABC method. Hematoxylin counterstaining. Scale bar, 100 μm.

In Kaplan-Meier Plotter, *KRT5* mRNA expression was associated with a poor prognosis of patients with serous ovarian carcinoma with TP53 mutation (n=470, Figure S1). According to scRNA-seq datasets, all HGSC cases (n=29) contained KRT5+ cells, and 48% (14 out of 29) HGSC contained at least 5% of KRT5+ cells (Table S2). We further confirmed KRT5 expression by immunostaining in 197 HGSC cases with known clinical outcomes (Figure 1). Higher frequency of KRT5+ cells was associated with significantly shorter survival after diagnosis (Figure 1 and Figure S2). Overall, these observations are consistent with the previous report based on 117 patients [23].

### 2.2. Isolation and characterization of KRT5+ cells

**Figure 2.**
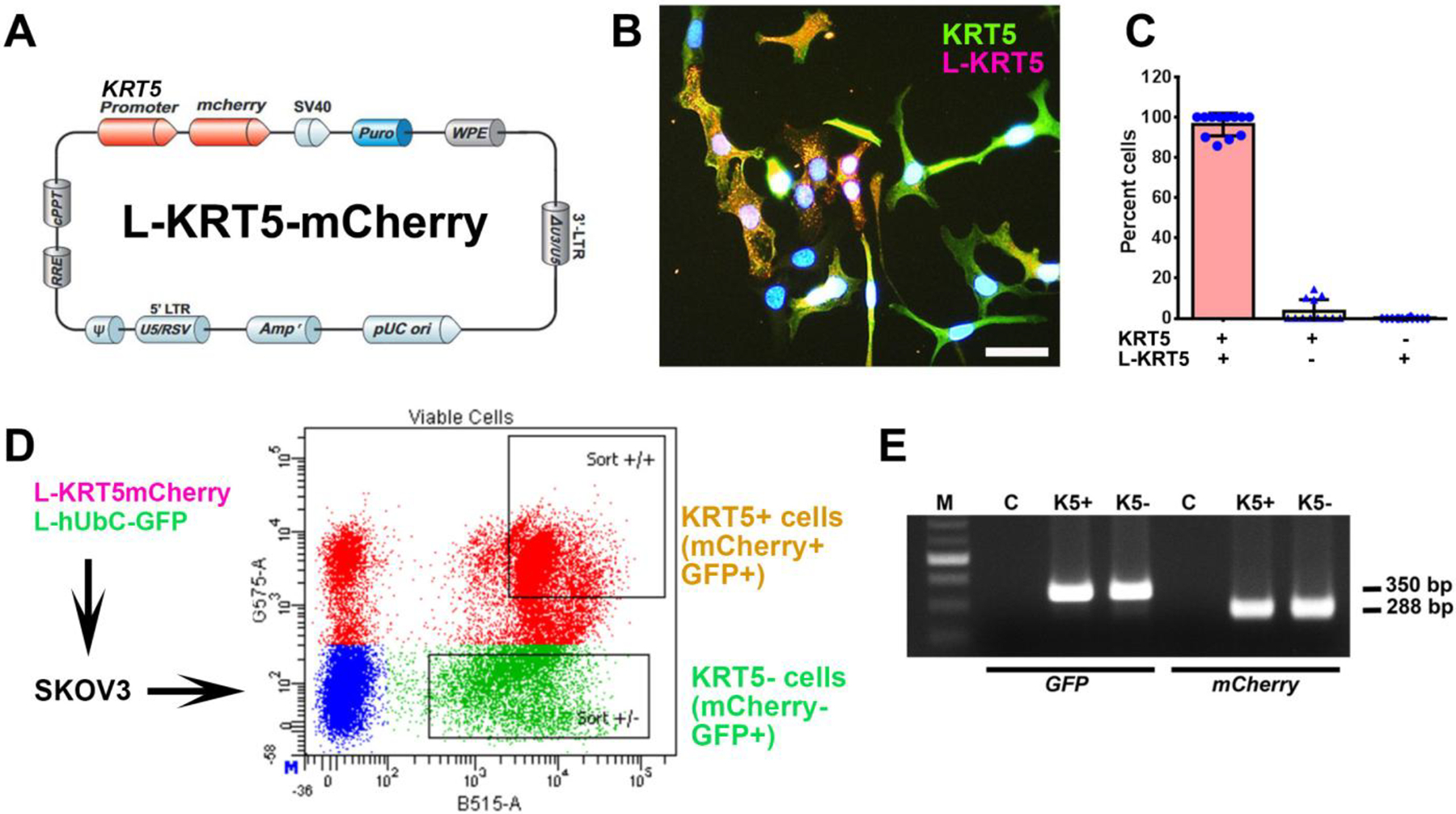
Isolation and characterization of KRT5+ and KRT5− cells from SKOV3 cell line. **(A)** Structure of Lenti-KRT5mCherry (L-KRT5mCherry). **(B)** Co-localization of KRT5 immunostaining (KRT5, green) and mCherry (L-KRT5) expression (magenta). Orange, overlay. Counterstaining with DAPI (blue). Scale bar, 60 μm. **(C)** Quantification of cells expressing both KRT5 and L-KRT5 (KRT5+ L-KRT5+), or only KRT5 (KRT5+) or L-KRT5+. **(D)** Experimental design of isolation of cells expressing both L-KRT5mCherry and L-hUbC-GFP or L-hUbC-GFP alone. **(E)** PCR detection of L-hUbC-GFP (GFP) and L-KRT5mCherry (mCherry) DNA in both KRT5+ (K5+) and KRT5− (K5−) cells. All error bars denote s.d.

To gain insights into the biological properties of KRT5+ cells, we transduced SKOV3 cells with lentiviruses containing *KRT5* promoter driving expression of reporter mCherry (Figure 2A–C). Over 95% of KRT5+ cells expressed mCherry (Figure 2B and 2C). No expression of mCherry was observed in KRT5− cells. To aid in identification of KRT5+ cell progenies irrespective of whether KRT5+ expression has been retained in daughter cells, cells were co-transduced with lentivirus expressing GFP driven by the ubiquitous *hUbC* promoter (L-hUbC-GFP, Figure 2D). We then isolated KRT5+ and KRT5− cell populations using fluorescence-activated cell sorting (FACS). According to PCR analysis, both lentiviruses were evenly integrated into both KRT5+ and KRT5− cells (Figure 2E). Thus, our approach provides a platform to test both KRT5+ and KRT5− cell populations for their functional properties.

**Figure 3.**
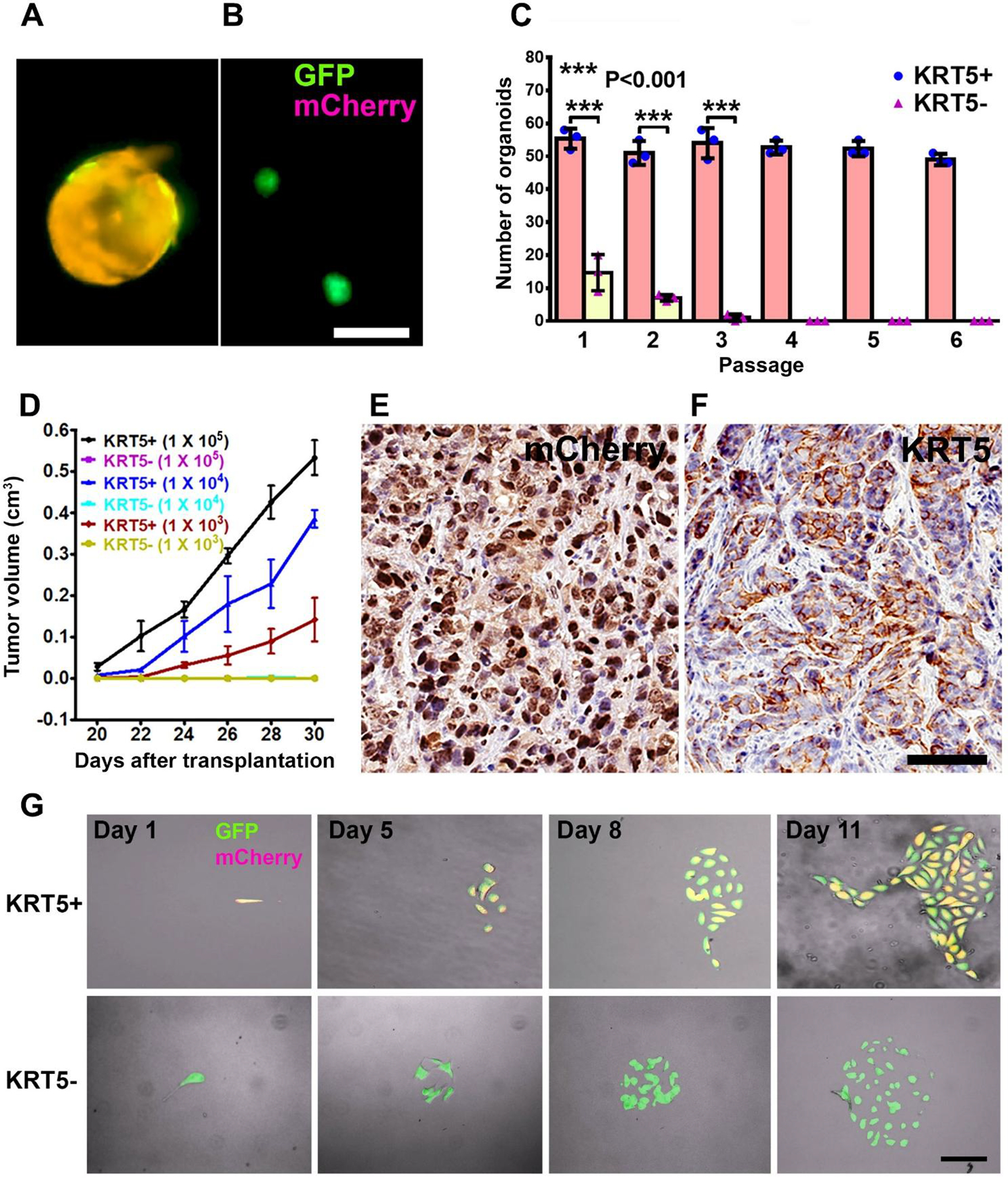
Characterization of KRT5+ cells. **(A**, **B)** Organoids derived from SKOV3 cells expressing both lentiviruses (A, GFP and mCherry, orange) or GFP alone (B, green). Scale bar, 100 μm. **(C)** Quantification of KRT5+ (blue symbols, pink bars) and KRT5− (pink symbols, yellow bars) cancer organoids in 6 consecutive passages. All error bars denote s.d. **(D)** Volume of tumors formed by serially diluted (1 × 10^5^, 1 × 10^4^, 1 × 10^3^) of KRT5+ and KRT5− cells after their s.c. transplantation into different flanks of NSG mice. KRT5− group did not form tumors. **(E**, **F)** mCherry (E) and KRT5 (F) expression in KRT5+ cell derived xenografts. Elite ABC method. Hematoxylin counterstaining. Scale bar, 60 μm. All error bars denote s.d. **(G)** Live microscopy of cells were isolated by FACS based on their expression of GFP (green) and mCherry (magenta) after coinfection with Lenti-UbC-GFP and Lenti-KRT5mCherry. Orange, Overlay. Individual frames of live microscopy. Scale bar, 60 μm.

KRT5+ cells successfully generated cancer organoids for 6 passages, with each passage lasting 12 days, following seeding in Matrigel. In contrast, KRT5− cells were only capable of forming organoids for up to three passages (Figure 3A–C). Compared with KRT5− cells, organoids derived from KRT5+ cells were significantly larger (Figure 3A and 3B) and formed at a higher frequency (Figure 3C). Similar results were obtained after growing organoids in ultra-low-attachment plates (Figure S3).

To assess the tumorigenicity potential of KRT5+ and KRT5− cells *in vivo*, FACS-isolated and serially diluted (1 × 10^5^, 1 × 10^4^, 1 × 10^3^) KRT5+ and KRT5− cells were subcutaneously transplanted into NOD scid gamma (NSG) mice. Beginning 20 days post-transplantation, KRT5+ cells formed tumors in a dose-dependent manner, whereas KRT5− cells failed to form tumors by 30 days after transplantation (Figure 3D; Table S3). Tumor tissues were collected at the experimental endpoint on day 30 and subsequently validated for mCherry and KRT5 expression by immunostaining (Figure 3E and 3F).

To test whether KRT5+ cells give rise to KRT5− cells and vice versa, we seeded single KRT5+ or KRT5− cells in a 35 mm μ-Dish with a glass bottom and followed their fate at 24-h intervals for 8–10 days using a fluorescence microscope (Figure 3G). KRT5+ cells gave rise to KRT5− cells (n=3 independent tracing experiments). However, KRT5− cells did not revert into KRT5+ cells (n=3 independent tracing experiments).

### 2.3. KRT5+ cells have increased resistance to anti-cancer drugs

**Figure 4.**
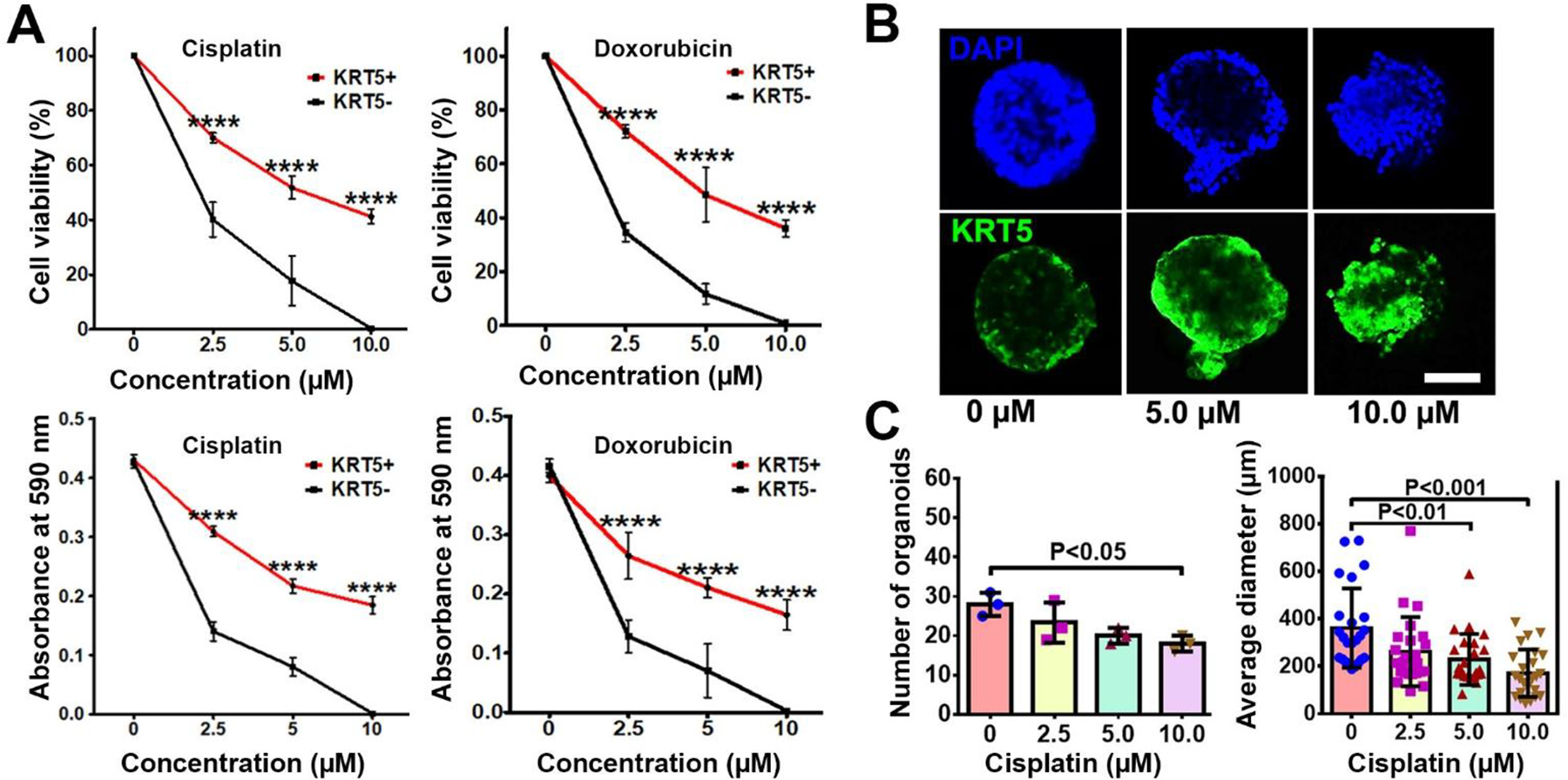
KRT5+ cells are chemoresistant to anti-cancer drugs. **(A)** Effect of cisplatin and doxorubicin on KRT5+ and KRT5− cells. Manual count (cell viability, upper row) and MTT assay (absorbance at 590 nm, bottom row) of KRT5+ and KRT5− cells after 72 h of treatment with cisplatin and doxorubicin at different concentrations. **(B, C)** Representative confocal images of primary HGSC organoids (B) and quantification of organoid frequency (C, left image) and size (C, right image) after treatment with different concentrations of cisplatin for 72 h. Immunofluorescence for KRT5 (green). Counterstaining with DAPI (blue). Scale bar, 50 μm for all images. All error bars denote s.d.

To compare chemoresistance of KRT5+ and KRT5− cells, they were treated with increasing concentrations (2.5 μM, 5.0 μM, and 10.0 μM) of common antineoplastic drugs, cisplatin and doxorubicin (Figure 4A). According to both manual counting and MTT assay, there was a significant decrease in the cell viability of both KRT5+ and KRT5− cells in a dose-dependent manner 72 h after treatment. However, KRT5+ cells were significantly more resistant, with approximately 35%–40% viable cells after treatment with the highest concentrations of either cisplatin or doxorubicin.

To confirm the translational relevance of our findings, we evaluated effect of drugs on KRT5 expression in three KRT5+ tumorigenic organoid systems established by us from individual HGSC samples. In agreement with observations on cancer cell lines, treatment of cancer organoids with increasing concentrations of cisplatin resulted in reduction of organoid numbers and their sizes (Figure 4B and 4C). At the same time, the relative number of KRT5+ cells increased.

### 2.4. Distinct gene expression signatures of KRT5+ and KRT5− cells

**Figure 5.**
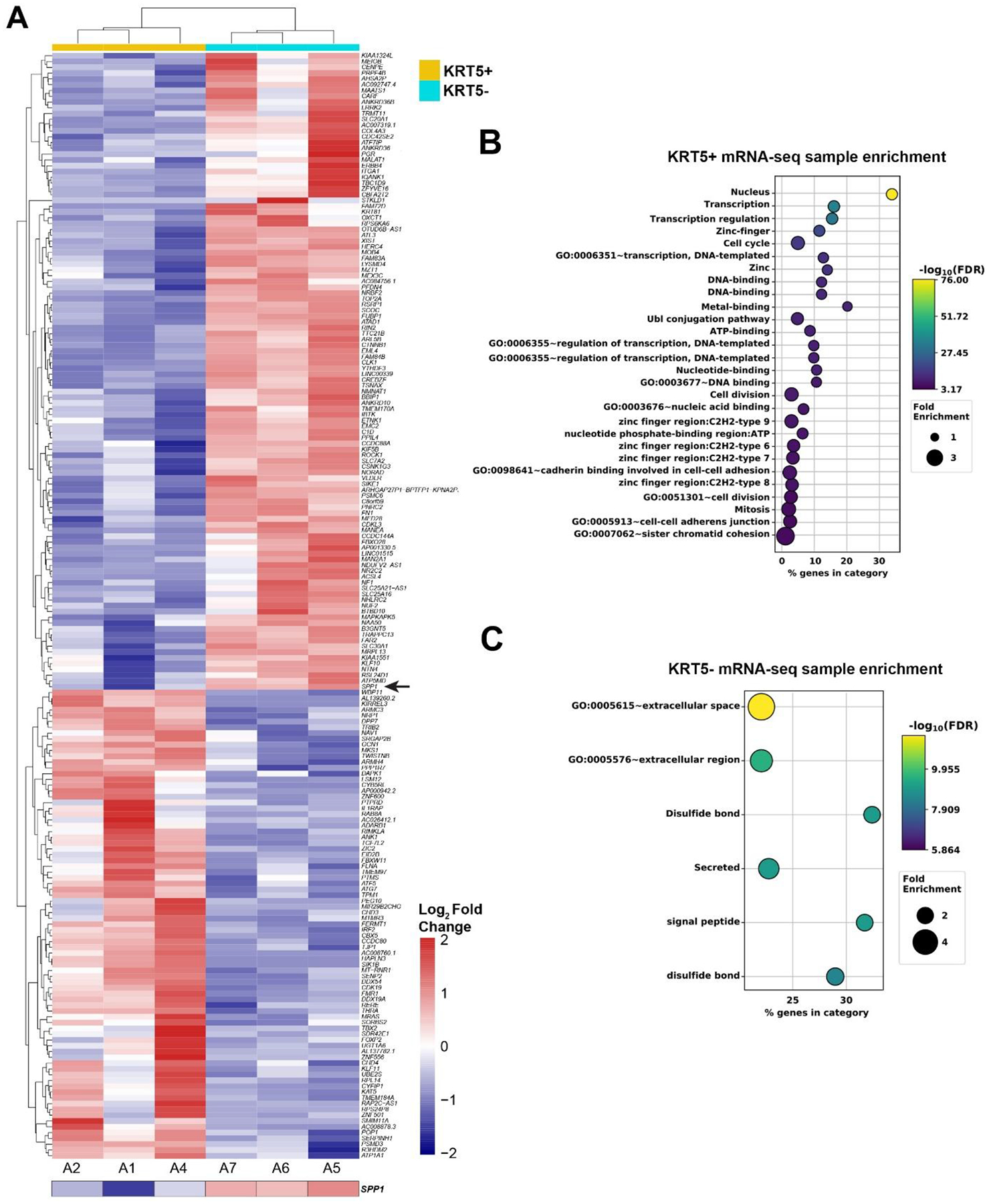
Gene expression analysis of KRT5+ and KRT5− cells isolated from SKOV3 cell line. **(A)** Heat map depicting the top 200 differentially expressed genes in KRT5+ (A2, A1 and A4) and KRT5− (A7, A6, and A5) cells. Note the expression of *SPP1* (osteopontin) in KRT5− cells (arrow). **(B,C)**, DAVID analysis for functional annotation of terms associated with KRT5+ (B) and KRT5− (C) cells.

To compare transcriptomes of KRT5+ and KRT5− cells, we sorted 3 independent samples by FACS followed by RNA sequencing and visualized the top 200 differentially expressed genes distinguishing KRT5+ and KRT5− groups (Figure 5A). According to DAVID bioinformatics analysis, KRT5+ cells’ transcriptomes were significant in transcription regulation, cell division/cycle, cell-cell adhesion, and DNA damage and repair annotations (Figure 5B). At the same time, levels of key drug efflux pumps (e.g., *ABCB1*, *ATP7B)* or importers (e.g., *SLC31A1*/*CTR1*) were not significantly different between KRT5+ and KRT5− cells (Figure S4). Functional GO enrichment of KRT5− cells revealed that the top significant terms were observed in secreted, signal peptide, and extracellular-related annotations (Figure 5C).

### 2.5. KRT5− cells produce OPN facilitating maintenance and chemoresistance of KRT5+ CPCs

**Figure 6.**
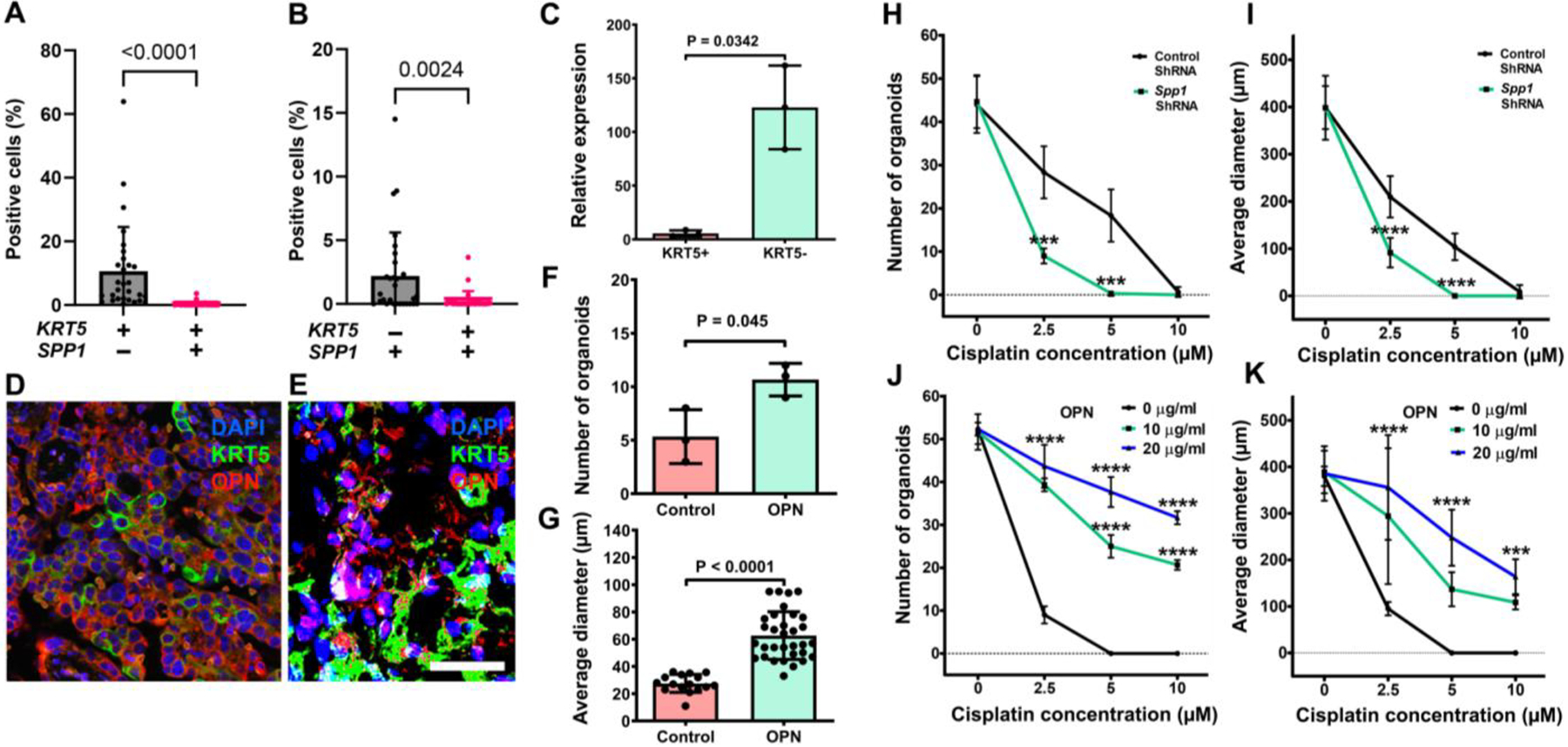
Characterization of the role of OPN in HGSC. **(A**, **B)** Quantification of cells expressing *KRT5* and/or SPP1 cells in human HGSC cases from single-cell RNA sequencing. Significance by Mann-Whitney U test. **(C)** RT-PCR analysis of *SPP1* expression in KRT5+ and KRT5− subpopulations of SKOV3 cells. **(D**, **E)** KRT5 (green) and OPN (red) expression in HGSC (D) and primary HGSC organoids (E). Double immunofluorescence, counterstaining with DAPI (blue). Scale bar: (D) 60 μm, E (40 μm). **(F**, **G)** OPN treatment increases frequency (F) and size (G) of HGSC organoids (n=3). **(H-K)** Effect of cisplatin on frequency (H and J) and size (I and K) of organoids either transduced with *SPP1* shRNA (H and I) or treated with OPN (I and K). All organoids were measured 72 h after treatment with cisplatin at different concentrations. All error bars denote s.d.

During RNA-seq analysis and screening for genes encoding proteins differentially secreted by CPCs *versus* non-CPCs cells, we found that *SPP1*, the gene encoding osteopontin (OPN), was highly expressed in KRT5^−^ cells (Figure 5A). These results were corroborated with publicly available single-cell RNA sequencing data from 41 patients with HGSC. Within these datasets, *KRT5* and *SPP1* co-expression was rarely reported, and a majority of *KRT5*+ epithelial cancer cells did not express *SPP1* and vice versa (Figure 6A and 6B).

Preferential *SPP1* expression in KRT5− cells was confirmed by RT-PCR of sorted cells (Figure 6C) and by immunostaining of primary HGSC samples (Figure 6D, n=8) and HGSC organoids (Figure 6E, n=3). Supporting the biological significance of these observations, addition of OPN increased HGSC organoid frequency and size (Figure 6F and 6G). Importantly, *SPP1* knockdown by shRNA potentiated effects of cisplatin on frequency and size of organoids (Figure 6H and 6I), whereas increased OPN concentration had opposite effects in a dose-dependent manner (Figure 6J and 6K).

We also have found that expression of key OPN receptor, *CD44*, is enriched in the KRT5+ CPCs’ fraction of RNA-seq dataset of SKOV3 cells (Figure S5). This suggests that the OPN-CD44 axis is an important signaling pathway.

## 3. Discussion

Heterogeneity in cancer cellular states is closely associated with the propagation of CPCs. Here, we identify KRT5 expression as a reliable marker of CPCs in HGSC. This observation is consistent with previous reports [22,23] and our current findings that HGSC tumors with a high proportion of KRT5−positive cells exhibit more rapid progression and increased resistance to chemotherapeutic agents.

KRT5 is a well-established marker of stem/progenitor cells in lung and prostate epithelia [25,26]. Its expression has also been detected in fallopian epithelial cells with stem-like properties, including the expression of epithelial cell adhesion molecule (EpCAM), CD44, and integrin α6, and the capacity for clonal growth and self-renewal in sphere formation assays [27]. To date, KRT5 expression has not been reported in serous tubal intraepithelial carcinoma. Notably, in mouse models, *Krt5* expression marks pre-ciliated tubal epithelial cells that are prone to HGSC initiation [28]. These findings raise important questions regarding the evolutionary relationship between normal and neoplastic KRT5+ cell populations, which remain to be elucidated.

Hierarchies of cellular differentiation and the associated interrelationship among the tumor cells may determine the overall growth of the tumors and their susceptibility to drugs and targeted therapeutic approaches [19,29–33]. Our study shows that HGSC contains two hierarchically related cell populations: KRT5+ CPCs and KRT5− non-CPCs. These cell populations have distinct transcriptome profiles that should allow in-depth evaluation of pathways associated with either tumorigenicity of CPCs or niche-producing factors in non-CPCs.

Non-CPCs produce OPN and thereby support growth and chemoresistance of CPCs by activating OPN signaling pathway. OPN interacts with cell surface receptors, such as CD44 and integrins, and plays an important role in a wide range of physiological and pathological processes, such as cell adhesion, proliferation, survival, and angiogenesis [34,35]. OPN signaling is known to regulate stem/progenitor cell proliferation and differentiation in liver, gastroesophageal cancer and hematopoietic systems, and also to promote stemness of melanoma, glioma and colon cancers [36–41]. OPN overexpression correlates with the worst prognosis in human gastric and gastroesophageal carcinomas [41,42].

OPN has been reported as a biomarker in ovarian cancers based on its detection in blood, primary and metastatic carcinomas [43–48]. It has been reported that acquired expression of OPN by HO-8910 cells greatly promotes soft agar colony formation and tumor growth *in vivo* [49]. Our study uncovers the roles of OPN as a local niche factor in HGSC, and its effects on stem cell properties and chemoresistance of ovarian cancer cells.

Our study shows that a key OPN receptor, *CD44* is preferentially expressed in KRT5+ cells in SKOV3 cells. CD44 is a polymorphic transmembrane glycoprotein that is expressed in multiple cell types [50]. This cell surface receptor participates in a wide variety of cellular functions, such as cell growth, differentiation, apoptosis, and motility [51,52]. CD44 is expressed by Lgr5+ stem cells in the intestinal crypts [53] and stem/progenitor cells in gastric squamous-columnar junction [41]. It is also used as the marker for mesenchymal stem cells and hematopoietic stem cells [54,55]. CD44 is expressed in KRT5+ human tubal epithelial with stem-like properties [27]. Future studies should address the degree of correlation between the prognostic significance of KRT5 and CD44 expression in HGSC. They are also expected to provide more mechanistic details and evaluate whether OPN-CD44 signaling is a potential target for preventive or therapeutic intervention in ovarian cancers. Since CD44 is a surface protein, its reliable detection in FFPE samples is more challenging than that of KRT5. Accordingly, KRT5 may be a more clinically applicable marker.

In this study, we used the ovarian cancer cell line SKOV3, which is highly valued for its experimental versatility and consistent tumorigenicity. Several publications have questioned whether SKOV3 accurately represents HGSC biology. A central argument in the widely cited paper by Domcke *et al*. [56] is that SKOV3 harbors wild-type *TP53*, in contrast to the near-universal *TP53* mutation status of HGSC. Because SKOV3 has also been reported to carry *ARID1A* and *PIK3CA* mutations, which are characteristic of clear-cell and endometrioid ovarian carcinomas, it has been proposed that this line may instead be derived from one of these histotypes [57,58]. However, multiple peer-reviewed studies explicitly support *TP53* loss-of-function/null status in SKOV3 [58–61]. SKOV3 also harbors RB pathway disruption, another hallmark of HGSC [59,62]. At the same time, large genomic studies, including TCGA, have shown that *ARID1A* and *PIK3CA* mutations can also occur in HGSC, albeit at low frequency [62, 63]. In addition, in the absence of access to the original primary tumor material, it cannot be excluded that some of these mutations arose ex vivo as a result of genetic drift or clonal evolution during cell culture [64].

Importantly, SKOV3 displays an immunophenotype that closely resembles HGSC [58,65] and has shown findings consistent with primary HGSC and other HGSC cell lines in recent studies [65,66]. Our results in SKOV3 cells are also consistent with observations from HGSC organoids, chemoresistance studies, and single-cell transcriptomic datasets. It should also be noted that even the most “accurate” HGSC cell lines, such as KURAMOCHI and OVSAHO, have limitations, including lack of KRT7 expression and limited tumorigenic potential [59]. Thus, although imperfect, SKOV3 falls within the spectrum of molecular and cellular properties, as well as clinical features, associated with HGSC.

In summary, our study points to the importance of non-CPCs in maintenance of CPCs. Combined targeting pathways that support stem-like (KRT5+ OPN-responsive CPCs) and niche (KRT5− non-tumorigenic OPN-producing non-CPCs) cell phenotypes may maximize the efficacy of HGSC therapies. Furthermore, this approach may reduce therapeutic doses of highly toxic anticancer drugs.

## 4. Methods

### 4.1. Study groups and clinical data

Kaplan-Meier Plotter [67] was used as a survival analysis tool to evaluate *KRT5* mRNA expression in 470 patient samples obtained from The Cancer Genome Atlas (TCGA, https://www.cancer.gov/ccg/research/genome-sequencing/tcga) and other publicly available sources. KRT5 immunohistochemical staining was performed on tissue arrays prepared at the Institute of Pathology, Ludwig Maximilians University, Munich, Germany. Patients’ ages ranged from 37 to 100 years, with a mean age of 66 years. All patients were diagnosed with HGSC. For each case, a representative paraffin block containing invasive cancer tissue was selected for immunohistochemical analysis.

### 4.2. Cell culture

Human ovarian cancer cell lines SKOV3 (ATCC, HTB-77), CAOV3 (ATCC, HTB-75), and CAOV4 (ATCC, HTB-76) were obtained from the American Type Culture Collection. The SKOV3 cells were cultured in McCoy’s 5A medium (Corning, 10-050-CV) supplemented with 10% fetal bovine serum (FBS, Sigma, 12306C), 4 mM L-Glutamine (Corning, 25-005-Cl) and 100 IU mL^−1^/100 μg mL^−1^ penicillin/streptomycin (PS, Corning, 30-002-Cl).

CAOV3 cell line was maintained in Dulbecco’s Modified Eagle Medium (DMEM, Corning, 15-018-CV) supplemented with 10% FBS, 4 mM L-glutamine and 100 IU mL^−1^/100 μg mL^−1^ PS. The culture conditions for SKOV3 and CAOV3 were 95% air, 5% carbon dioxide (CO_2_), at a temperature of 37°C. The CAOV4 cell line was maintained in a Leibovitz’s L-15 (Corning, 10-045-CV) growth medium supplemented with 100 IU mL^−1^/100 μg PS in 100% air, at a temperature of 37°C. HEK293T (packaging cells, human kidney, ATCC, CRL-3216) cells were cultured in DMEM supplemented with 10% FBS, 1% non-essential amino acids (NEAA, Thermo Fisher, 11140-050), 1 mM sodium pyruvate (Corning, 25-000-Cl), in 95% air, 5% CO_2_, at 37°C. Cell lines were authenticated in-house and tested for *Mycoplasma* contamination every 6 months. Cell lines were used within 20 passages of thawing.

### 4.3. Lentiviral packaging, transduction and shRNA mediated knockdown experiments

We performed lentiviral packaging and transduction following previously described methodology [68]. Briefly, for lentivirus packaging, psPAX2 (Addgene, 12260), pMD2.G envelope plasmid (Addgene, 12259), and KRT5 promoter clone (GeneCopoeia, HRPM15909-LvPM02, mCherry) were employed. HEK293T cells were transfected with 10 μg KRT5 promoter clone, 7.5 μg psPAX2 and 2.5 μg pMD2.G using Lipofectamine 2000 (Thermo Fisher, 11668027) following the manufacturer’s instructions. To differentiate between KRT5+ and KRT5− cell populations, another aliquot of transfection mixture containing ubiquitous promoter that drives GFP (hUbC, Addgene, 14883), was prepared in the same way. Each transfection mixture was added to a culture plate containing at least 3 × 10^5^ HEK293T cells. The culture plates were incubated for 12 h, after which an additional 1 mL of antibiotic-free DMEM medium was added. Lentiviral supernatants were harvested after 48 h and concentrated using centrifugal filters (Amicon, Ultra-15, UFC903024). Freshly prepared lentiviral particles were used to transduce SKOV3 cells. Four days post-transduction, viral medium was replaced with SKOV3 growth medium containing 1 μg mL^−1^ puromycin to select for KRT5+ (mCherry+GFP+) and KRT5− (mCherry−GFP+) cell populations. Selected cells were cultivated for two passages, and either frozen in cryopreservation medium for storage at −80°C or immediately used for downstream applications. For gene silencing experiments, commercially available lentiviral particles with a pool of OPN (*Spp1*) shRNA (Santa Cruz Biotechnology, sc-36129-V) and scrambled control shRNA (Santa Cruz Biotechnology, sc-108080) were used to knock down *Spp1* expression in SKOV3 cells and patient-derived cancer organoids.

### 4.4. Fluorescence-activated cell sorting

For separation of KRT5+(mCherry+GFP+) and KRT5− (mCherry-GFP+) cell populations, the transduced SKOV3 cells were placed in Fluorescence-activated (FACS) cell sorting buffer (Phosphate buffered saline pH 7.4 (PBS), Corning 21-031-CV, + 1% bovine serum albumin, (BSA), Sigma A9647). Cell sorting and subsequent data analysis were performed on a FACS Aria II sorter that is equipped with the FACS DiVa software (BD Bioscience). Dead cells were excluded with Sytox Red (Thermo Fisher Scientific, S34859) and un-transduced SKOV3 cells served as controls. The brightest KRT5+ (mCherry+GFP+) and KRT5− (mCherry−GFP+) were identified and gated electronically based on their characteristic light-scattering properties on the mCherry and GFP channel emission pattern. The two populations were collected in two different tubes containing FACS cell sorting buffer and further stored at −80°C or immediately used for downstream applications. Fragments of L-KRT5mCherry (mCherry) and L-hUbC-GFP (GFP) DNA were detected by PCR with primers shown in Table S4.

### 4.5. Single cell tracing

KRT5+ (mCherry+GFP+) and KRT5− (mCherry-GFP+) cell populations from SKOV3 cell line were suspended in different tubes containing culture medium and further counted to use them for single cell tracing experiment. Medium containing cells was diluted to contain a single cell per 0.5 mL culture medium and seeded in a 35-cm glass-bottom dish. Cells were incubated at 37°C and 5% CO_2_. After attachment, the differentiation of a single cell was traced for 10 days using a fluorescent microscope that was equipped with mCherry and eGFP channels (Zeiss Axiovert 200 Live-Cell Incubation Fluorescence Microscope). The imaging data were processed using ImageJ and a movie was compiled for each KRT5+ and KRT5− cell.

### 4.6. Cancer organoid culture

Primary ovarian tumors were acquired immediately during the surgery, and a part of the tumor was collected for organoid culture as described previously [69] or fixed immediately in 4% paraformaldehyde for later histological assessment. For organoid culture initiation the cryopreserved tissues were thawed in vials for 2–3 min in a 37°C water bath. Afterwards, in sterile conditions, washed three times in PBS, placed into 2 mL 2D media (Gibco Advanced DMEM/F12, Thermo Fisher Scientific, 12634028) supplemented with 5% FBS, 12 mM Hepes (Thermo Fisher Scientific, 15630080), 1% L-GlutaMax-I (Thermo Fisher Scientific, 35050079), 100 IU mL^−1^/100 μg mL^−1^ PS, 10 μM Rho Kinase inhibitor (Y-27632, ROCKi, 1 mM, Millipore, 68800), 10 ng mL^−1^ epidermal growth factor human (EGF, 10 μg mL^−1^, Sigma, E9644) and then minced into 0.1 mm pieces using surgical blades. The minced tissues were collected into 10 mL PBS, centrifugated at 300×g for 7 min at +4°C. The supernatant was discarded and the cell pellet suspended in 10 mL digestion buffer (0.5 mg/mL collagenase type I (Gibco 17100-017, in Advanced DMEM/F12, 12 mM Hepes, 3 mM CaCl_2_) followed by 45 min incubation at 37°C in a water bath. After the incubation 30 mL of 2D medium was added to the 10 mL mixture, and the solution was passed through 100 μm (Falcon; #352360), 70 μm (Falcon; #352350), and 40 μm cell strainers (Falcon; #352340). Next, the suspension was centrifuged at 300×g for 7 min at +4°C. The cell pellet was suspended in 2D media, counted and adjusted to 5 × 10^4^ cells per rim assay. Matrigel (Corning; 356231) was added to a final concentration of Matrigel at 70%. Rim assays were seeded in rims of 24 well plates and cultures performed as previously reported [69].

FACS sorted SKOV3 cell line-derived KRT5+ and KRT5− cells were suspended in 2D media so that the suspension contains 5 × 10^4^ cells. Subsequently, organoid cultures were performed as previously described [69].

### 4.7. Cell viability assays

Cell viability after administration of either cisplatin (cis-ciammineplatinum(II) dichloride; Pt(NH_3_)_2_Cl_2_, Sigma, P4394) or doxorubicin (Sigma, 44583) was assessed by manual counting and 3-(4,5-dimethylthiazol-2-yl)-2,5-diphenyltetrazolium bromide (MTT) assays. For manual counting, cells were seeded into 6-well plates at a density of 5.0 × 10^4^ cells per well. The MTT assay was carried out as per the manufacturer’s instructions (Abcam, #ab211091). Briefly, cells were seeded at a density of 5.0 × 10^4^ cells/well in 96-well plates. The medium was discarded from cell cultures and 50 μL of serum-free medium and 50 μL of MTT solution were added to each well. The plates were incubated at 37°C for 3 h. After incubation, 150 μL of MTT solvent was added to each well, and incubated on an orbital shaker for 15 min, after which the absorbance was read at OD = 590 nm. All experiments had a minimum of three replicates.

### 4.8. Immunohistochemistry and quantitative image analysis

Immunohistochemical analysis of paraformaldehyde-fixed paraffin embedded tissues was performed using a modified ABC technique [70–72]. Briefly, the ovarian cancer patient-derived tumors and mouse xenografts were excised and fixed with 4% paraformaldehyde and incubated overnight at 4°C. Tissues were dehydrated and embedded in paraffin, sectioned at 5 μm thickness and subjected to IHC staining. The slides were deparaffinized in xylene and rehydrated over a graded ethanol series, and antigen retrieval was performed using citrate buffer at pH 6.0. All primary and secondary antibodies for immunohistochemistry are shown in Table S5. The VECTASTAIN ABC reagent (Vector Laboratories, PK-6100) and 3,3’-diaminobenzidine (DAB, Sigma, 1029240001) were used for signal detection. Tissue sections with no primary antibody served as negative controls. Tissues sections with immunoperoxidase staining were scanned and digitized using ScanScope CS2 (Leica Biosystems, Vista, CA, USA) with a 40× objective. Immunofluorescence-stained sections were mounted in ProLong Diamond Antifade Mountant with DAPI reagent (Thermo Fisher, P36962) and sealed using nail polish and further scanned using ScanScope FL (Leica Biosystems) with a 20× objective. Further, ImageJ software (National Institute of Health, Bethesda, MD, USA) was used for quantitative analysis of immunohistochemistry and immunofluorescence images as previously described [70–72].

For immunocytochemistry in cell culture, cells were grown on coverslips and fixed using 4% paraformaldehyde in PBS (pH 7.4) for 10 min at room temperature. Permeabilization was performed using PBS containing 0.1% Triton X-100 for 10 min, and washed in PBS three times for 5 min. The permeabilized cells were incubated with 1% BSA and 22.52 mg/mL glycine in PBST (PBS+ 0.1% Tween 20) for 30 min to block nonspecific binding. The cells were then incubated with the primary antibody overnight at 4°C. The cells were washed three times with PBS for 5 min and incubated with the secondary antibody for 1 h at room temperature in the dark. Primary and secondary antibodies used to stain cells are listed in Table S5. Stained cells were mounted in the same way as immunofluorescence-stained tissue sections. Confocal images were acquired using a Zeiss LSM 710 confocal microscope through the Cornell University Biotechnology Resource Center. The image data were merged and displayed with the ZEN software (Zeiss).

### 4.9. Quantitative real-time PCR

RNA was extracted using TRIzol reagent according to the manufacturer’s instructions (Thermo Fisher, 15596026). cDNA was produced using the SuperScript III First-Strand Synthesis kit (Thermo Fisher, 18080400). Real-time PCR was performed using PerfeCTa SYBR Green Super Mix Reagent (Quanta Biosc., 95072-250) on C1000 Touch Thermal Cycler PCR machine (Bio-Rad).

### 4.10. Tumorigenicity assays

FACS-isolated KRT5+ and KRT5− cancer cells from the transduced SKOV3 cell line were suspended in PBS and injected subcutaneously (5 × 10^5^, 5 × 10^4^, and 5 × 10^3^ cells in 250 μL) into either flank of 6–8-week-old *NOD.Cg-Prkdc*^*scid*^*Il2rg*^*tm1Wjl*^/*SzJ4* female mice. Control mice received only PBS. The size of the subcutaneous mass was measured using scientific caliper every alternate day starting from day 20 after transplantation until day 30. The tumor volume was calculated using VT = 1/2(L × W × H) and tumor incidence was also noted. Mice were euthanized and subjected to necropsy as soon as they showed signs of sickness such as abdominal distension, subcutaneous masses approximately 1 cm diameter on either flank or moribund behavior.

### 4.11. RNA sequencing

Total RNA was isolated using TRIzol reagent according to the manufacturer’s instructions. RNA integrity and concentrations were determined using a Nanodrop spectrophotometer and only the purified RNA samples were used for further analysis. ERCC Spike-In Mix 1 (Thermo Fisher, 4456740) was added to samples prior to library creation according to the manufacturer’s recommendation. 3’ mRNA-seq libraries were created using QuantSeq 3’ mRNA-Seq Library Prep Kit (New England Biolabs) according to the manufacturer’s instructions using 10 ng total RNA. The sequencing files were inspected for quality control analysis using FASTQC [73]. Reads were then aligned to the GRCh38 human reference genome using the STAR two-pass method. R (https://www.r-project.org/) was used for all analyses and visualization of aligned data. Differential gene-expression analysis was performed using the R package DESeq2 (V 1.6.3). A false discovery rate of ≤0.05 was then used to identify differentially expressed genes between sample group comparisons. Heat maps were generated using normalized average gene-count values within the groups. Only the genes that were differed significantly (*P* < 0.05) between KRT5+ and KRT5− groups were taken into consideration for further analysis. Furthermore, the Database for Annotation, Visualization and Integrated Discovery (DAVID) (v6.8) gene functional annotation and classification tool [74,75] was used to annotate the list of differentially expressed genes with the corresponding GO terms and to perform GO enrichment analysis and gene functional clustering for biological functions including significant pathways and gene ontology terms. Statistically significant signaling pathways were then identified by using a *P* value cutoff of 0.05.

### 4.12. Single-cell RNA sequencing analysis

A publicly available dataset for 29 HGSC cases [GSE180661] [76] was downloaded and aligned to the GRCh38 human reference genome using Cell Ranger (v7.1.0, 10x Genomics). All further preprocessing and data analysis were conducted in R (v4.2.0). The count matrices were processed using the standard SoupX (v.1.6.1) pipeline to remove ambient RNA signal (https://github.com/constantAmateur/SoupX). The corrected matrices were then processed in Seurat (v.4.3.0) (https://github.com/satijalab/seurat) to subset epithelial cells and quantify cell populations based on *KRT5* and *SPP1* expression.

### 4.13. Statistical analysis

Statistical analysis was performed using GraphPad Prism 10.6.1. All *in vitro* experiments were performed in biological triplicates unless otherwise stated. A two-tailed Mann-Whitney test and an unpaired *t*-test with Welch’s correction were used to compare two groups. ANOVA was conducted for multiple comparisons, followed by a post hoc Tukey test to identify differences between groups. All data are shown as the mean with SDs from at least three independent experiments. *P* > 0.05 were considered not significant, and significance was established as *, *P* < 0.05; **, *P* < 0.01; ***, *P* < 0.001; ****, *P* < 0.0001. Survival analysis was conducted using the Kaplan–Meier method. *P* < 0.05 indicated significant difference.

## Supplementary Material

SM Table S1. Frequency of KRT5+ cells in HGSC cell lines according to immunostaining.
Table S2. Frequency of KRT5+ cells in primary HGSC according to scRNA seq datasets.
Table S3. Tumor incidence.
Table S4. Genotyping primers.
Table S5. Antibodies used for immunostaining.
Figure S1. Kaplan–Meier analysis of overall survival in 470 patients with TP53-mutant serous ovarian carcinoma stratified by KRT5 mRNA expression.
Figure S2. Kaplan-Meier overall survival analysis of HGSC patients.
Figure S3. Characterization of cancer organoids derived from KRT5+ and KRT5− cells cultured in ultra-low-attachment plates.
Figure S4. Expression of key transporters in *KRT5+* SKOV3 cells.
Figure S5. *CD44* expression in *KRT5+* and KRT5− SKOV3 cells.

The following supplementary materials are available on the website of this paper at CHP2603020005SupplementaryMaterials.zip.

## Data Availability

The bulk RNA-seq data reported in this paper are deposited in Gene Expression Omnibus (GEO) under accession number GSE316118. The Source Data provides data for all results requiring quantification. Any additional data supporting the findings of this study are available from the corresponding author upon reasonable request.

## Abbreviations

The following abbreviations are used in this manuscript:

CPCs: Cancer propagating cells
DAPI: 4’,6-diamidino-2-phenylindole
EpCAM: epithelial cell adhesion molecule
FACS: Fluorescence activated cell sorting
HGSC: High -grade serous carcinoma
KRT5: Keratin 5
non-CPCs: non-cancer-propagating cells
NSG: NOD scid gamma
OPN: Osteopontin
